# Towards equity: a retrospective analysis of public sector radiological resources and utilization patterns in the metropolitan and rural areas of the Western Cape Province of South Africa in 2017

**DOI:** 10.1186/s12913-021-06997-x

**Published:** 2021-09-20

**Authors:** Beulah Christina van Zyl, Michelle Monique Barnard, Keith Cloete, Amanda Fernandez, Matodzi Mukosi, Richard Denys Pitcher

**Affiliations:** 1 Division of Radiodiagnosis, Department of Medical Imaging and Clinical Oncology, Faculty of Medicine and Health Sciences, Stellenbosch University and Tygerberg Hospital, Francie van Zijl, Avenue, Tygerberg, Cape Town, 7505 South Africa; 2 Sub-Directorate Medical Imaging Services, Directorate: Health Technology, Western Cape Department of Health, 1st Floor North Block, Bellville Health Park, c/o Mike Pienaar Boulevard & Frans Conradie Drive, Bellville, Cape Town, 7500 South Africa; 3 Department of Health, Western Cape Government, Cape Town, South Africa

**Keywords:** Radiology resource utilization, Equity, Universal health coverage, MIC

## Abstract

**Background:**

The reduction of inequality is a key United Nations 2030 Sustainable Development Goal (WHO, Human Resources for Health: foundation for Universal Health Coverage and the post-2015 development agenda, 2014; Transforming our world: the 2030 Agenda for Sustainable Development .:. Sustainable Development Knowledge Platform, 2020). Despite marked disparities in radiological services globally, particularly between metropolitan and rural populations in low- and middle-income countries, there has been little work on imaging resources and utilization patterns in any setting (Transforming our world: the 2030 Agenda for Sustainable Development .:. Sustainable Development Knowledge Platform, 2020; WHO, Local Production and Technology Transfer to Increase Access to Medical Devices, 2019; European Society of Radiology (ESR), Insights Imaging 6:573-7, 2015; Maboreke et al., An audit of licensed Zimbabwean radiology equipment resources as a measure of healthcare access and equity, 2020; Kabongo et al., Pan Afr Med J 22, 2015; Skedgel et al., Med Decis Making 35:94-105, 2015; Mollura et al., J Am Coll Radiol 913-9, 2014; Culp et al., J Am Coll Radiol 12:475-80, 2015; Mbewe et al., An audit of licenced Zambian diagnostic imaging equipment and personnel, 2020). To achieve equity, a better understanding of the integral components of the so called “imaging enterprise” is important. The aim was to analyse a provincial radiological service in a middle-income country.

**Methods:**

An institutional review board-approved retrospective audit of radiological data for the public healthcare sector of the Western Cape Province of South Africa for 2017, utilizing provincial databases.

We conducted population-based analyses of imaging equipment, personnel, and service utilization data for the whole province, the metropolitan and the rural areas.

**Results:**

Metropolitan population density exceeds rural by a factor of ninety (1682 vs 19 people/km^2^). Rural imaging facilities by population are double the metropolitan (20 vs 11/10^6^ people). Metropolitan imaging personnel by population (112 vs 53/10^6^ people) and equipment unit (1.7 vs 0.7/unit) are more than double the rural. Overall population-based utilization of imaging services was 30% higher in the metropole (289 vs 214 studies/10^3^ people), with mammography (24 vs 5 studies/10^3^ woman > 40 years) and CT (21 vs 6/10^3^ people) recording the highest, and plain radiography (203 vs 171/10^3^ people) the lowest differences.

**Conclusion:**

Despite attempts to achieve imaging equity through the provision of increased facilities/million people in the rural areas, differential utilization patterns persist.

The achievement of equity must be seen as a process involving incremental improvements and iterative analyses that define progress towards the goal.

## Background

The reduction of inequality is a key United Nations 2030 Sustainable Development Goal (SDGs) [1, 2]. Diagnostic imaging is recognised as a key building block of any healthcare system and is considered essential for effective primary care [3–12]. However, radiological services are expensive, labour-intensive, require high levels of technical expertise and are thus amongst the leading drivers of escalating medical costs [13–17].

The expense of modern diagnostic imaging has the potential to compound existing worldwide inequalities in access to radiological services. At one end of the spectrum are high-income countries with an aging population, a high burden of non-communicable diseases and a relative abundance of the more capital intensive imaging equipment such as mammography, computerised tomography (CT), magnetic resonance imaging (MRI) and digital subtraction angiography (DSA) [13, 18]. At the other end are more than half the world’s population, living in low- and middle-income countries (LMIC) by World Bank criteria, with diseases predominantly related to poverty, and with only limited access to basic and affordable imaging equipment such a plain X-ray (XR) and ultrasound (US) [7, 9, 13, 19]. There are marked disparities in radiological equipment resources amongst countries in the same World Bank economic grouping, as well as between geographical regions and healthcare sectors within the same country. The greatest divide is between metropolitan and rural populations [2, 7, 11, 20–25].

There has been limited in-depth work on radiological resources and service utilization patterns globally, particularly in LMICs. Although the WHO has published national estimates of high-end medical imaging resources based on questionnaire surveys of member countries, these data do not include the more basic and affordable equipment [19]. Furthermore, while Stellenbosch University in Cape Town, South Africa, is coordinating a project to collate detailed data on registered radiological resources in Southern, East and West Africa, these data do not include ultrasound equipment or utilization data [20, 21, 25, 26]. Despite the European Commission conducting detailed surveys of the use of ionizing radiation for medical purposes, data are not correlated with imaging equipment and personnel resources [27]. Although there has been an analysis of differential geographical utilization of radiological services in Norway [28], a high-income country, the need exists for such analyses in less resourced settings, incorporating all components of the so-called “imaging enterprise” [16]. The healthcare infrastructure of the Western Cape Province (WCP) of South Africa (SA) is ideal for such an analysis.

SA is one of five upper middle-income countries in sub-Saharan Africa. It has adopted the District Health System (DHS) for delivery of comprehensive primary care in the public healthcare sector [29]. Health services are devolved to the country’s nine provinces. The WCP is SA’s southernmost province, with six administrative districts. The City of Cape Town Metropolitan District is surrounded by five sprawling rural Districts [30], in which the main economic activities are agriculture and recreation.

Approximately three quarters (75.3%) of the WCP population have no medical insurance and are thus dependent on public sector health services, which are managed along geographic lines, and stratified as *metropolitan* or *rural* streams [31]. Parallel, tiered referral pathways exist in each system. First access to imaging is generally at Clinics or Community Day Centres, staffed by nursing personnel and allied health professionals, including radiographers, who provide basic out-patient care during office hours. Community Health Centres are staffed by medical officers, nurses and radiographers who provide a more advanced level of basic outpatient care, including limited after-hour services. Sequential referral is to District, Regional, and Central Hospitals, with incremental levels of 24-h in- and out-patient services, the latter being university-affiliated tertiary teaching hospitals. Rural referral patterns from District to Regional Hospitals cross individual district boundaries.

The WCP has a provincial-wide digital imaging platform, with picture archiving and communication system (PACS)-integration of services at the various levels of care. This facilitates access to all images across the platform and eliminates unnecessary duplication of investigations. Clinicians at lower tiers of service can also get assistance in interpretation of investigations via the digital platform.

The Medical Imaging Services Sub-Directorate within the Directorate of Health Technology in the WCP Department of Health (DoH) is responsible for stringent collation of all data pertaining to provincial imaging resources. These data include the number, location and functionality of all radiological equipment units, the number and placement of all imaging personnel, as well as the utilization of services at each facility. Such data provide unique insights into public sector imaging services in a middle-income country.

The aim of this study was to analyse the resources and utilization patterns of the WCP public sector radiological platform, including those features differentiating metropolitan from rural services.

## Objectives

1. Define the number of WCP public sector equipment units/10^6^ people by radiological modality in 2017, for the province as a whole, the metropolitan and rural areas
2. Define the number of WCP public sector imaging personnel/10^6^ people by category of healthcare worker in 2017, for the province as a whole, the metropolitan and rural areas.
3. Define the number of WCP public sector radiological examinations/10^3^ people by modality in 2017, for the province as a whole, the metropolitan and rural areas
4. Define the number of WCP public sector radiological examinations/10^3^ patients by modality in 2017, for the province as a whole, the metropolitan and rural areas
5. Define the average number of WCP examinations/equipment unit by modality in 2017, for the province as a whole, the metropolitan and rural areas.
6. Assess the combined contribution of plain X-ray and ultrasound studies to the total provincial workload.

### Hypothesis

Differential radiological resource utilization patterns exist between metropolitan and rural areas of the WCP.

## Methods

This was a retrospective audit of diagnostic imaging data for the public healthcare sector of the WCP of SA for 2017. Private imaging resources were excluded from the analysis since routine access to such services is limited to the insured population. All radiological details were extracted from the databases of the Medical Imaging Services Sub Directorate of the Directorate of Health Technology of the WCP DoH. Population particulars were based on Stats SA District Council Projections for 2017 [29].

Data on radiological equipment units and examinations performed were captured on a customized spreadsheet and stratified by imaging modality [XR, US, CT, whole body radiography (LODOX), fluoroscopy, mammography, MRI and DSA], healthcare facility and provincial service stream (metropolitan/rural). All equipment units included in the study were fully functional and serviced regularly. The single metropolitan stream consists of the City of Cape Town, while the parallel rural stream includes five districts namely West Coast, Cape Winelands, Overberg, Garden Route and Central Karoo. Rural referral pathways from District to Regional Hospital breach individual district boundaries, precluding analysis by individual rural district [30]. Plain radiography and fluoroscopy units were further subdivided into fixed and mobile units. Plain radiographic examinations were categorised as chest radiographs (CXR) and general radiographs (GR) the latter being all plain radiographs other than CXRs. Total radiographs (TR) denoted the sum of all CXR and GR examinations. All analyses were for the province as a whole and for the metropolitan and rural areas.

The number of radiology equipment units per million people was calculated by modality. The quantum of radiological examinations for each modality was assessed per thousand people, as well as per thousand patient engagements. Imaging examinations per equipment unit were calculated by modality.

The numbers of registered personnel in the categories of diagnostic radiographer, sonographer, registrar, and specialist radiologist were collated and categorized by healthcare facility and service stream.

The study was approved by the Head of Health of the Western Cape Government (WCG), the WCP Health Research Committee, under the auspices of the National Health Research Database and the Health Research Ethics Committee of Stellenbosch University.

## Results

### Provincial overview

Approximately two-thirds of the WCP population live in the City of Cape Town Metropolitan District which constitutes just 2 % of overall provincial land area. Metropolitan population density (1682 people/km^2^) exceeds rural (19 people/km^2^) by a factor of almost ninety (89:1) (Table 1).

**Table 1:** WCP radiological resources

	Rural	Metropolitan	Western Cape Province (WCP)
**Population (n)**			
Total (%)	2,366,893 (37)	4,114,404 (63)	6,481,297
Persons dependent on public health care	1,782,270	3,098,146	4,880,417
Women > 40 years dependant on public health care	291,138	509,695	800,833
Patients (per 10^3^ people)	282,220 (158)	714,093 (230)	996,313 (204)
**Geography**			
Area (km^2^)	127,018	2446	129,464
Population density (people/km^2^)	19	1682	50
**Facilities with an imaging service per 10**^**6**^**people)**	**35 (20)**	**33 (11)**	**68 (14)**
Central Hospital	0	3	3
Regional Hospital	3	3	6
District Hospital (% of facilities)	26 (74.0)	8	34 (50.0)
Community Health Centre	0	10	10
Community Day Centre (% of facilities)	5	9 (27.0)	14 (21.0)
Clinic	1	0	1
**Personnel n (per 10**^**6**^**people)**			
Radiologist	5 (3.0)	25 (8.1)	30 (6.1)
Registrar	N/A	44 (14.2)	44 (9.0)
Radiographer	79 (44.3)	260 (83.9)	339 (69.5)
Sonographer	11 (6.2)	19 (6.1)	30 (6.1)
**Total**	**95 (53.3)**	**348 (112.3)**	**443 (90.8)**
**Equipment n (per 10**^**6**^**people)**			
Fixed x-ray	44 (24.7)	64 (20.7)	108 (22.1)
Mobile x-ray	26 (14.6)	40 (12.9)	66 (13.5)
**Total x-ray**	**70 (39.3)**	**104 (33.6)**	**174 (35.7)**
Ultrasound	44 (24.7)	45 (14.5)	89 (18.2)
Fixed fluoroscopy	3 (1.7)	9 (2.9)	12 (2.5)
C-arm fluoroscopy	13 (7.3)	23 (7.4)	36 (7.4)
**Total fluoroscopy units**	**16 (9.0)**	**32 (10.3)**	**48 (9.8)**
Mammography units (per 10^6^ women > 40 years)	3 (14.0)	2 (5.0)	5 (8.0)
CT	3 (1.7)	10 (3.2)	13 (2.7)
MRI	0	3	3 (0.6)
Lodox	0	3	3 (0.6)
Angiography	0	9	9 (1.8)
Panoral	3	2	5
**Total units**	**139 (78.0)**	**210 (67.8)**	**349 (71.5)**

### Imaging facilities

There are sixty-eight (*n* = 68) provincial imaging facilities. All have plain radiography and fifty-three (*n* = 53/68; 78%) have ultrasound services. The metropolitan and rural areas have 35 and 33 imaging facilities, respectively. As a result, rural resources by population are almost double the metropolitan (20 vs 11/10^6^ people) (Table 1).

District Hospitals (*n* = 34/68, 50%) and Community Day Centres (*n* = 14/68, 21%) together account for almost three-quarters (*n* = 48/68, 71%) of all provincial facilities, with Community Day Centres predominant (*n* = 9/33, 27%) in the metropole and District Hospitals (*n* = 26/35, 74%) most common in the rural areas. All Central Hospitals (*n* = 3) and Community Health Centres (*n* = 10) are in the metropole, while the six Regional Hospitals are equally distributed between the metropolitan and rural areas (Table 1).

### Imaging equipment

Of the 349 provincial equipment units, plain radiography units constitute half (*n* = 174/349; 50%), ultrasound machines approximately a quarter (*n* = 89/349; 26%) and CT scanners less than 5 % (*n* = 13/349; 4%) (Table 1).

There are 36 radiography and 18 ultrasound units per million people overall. However, the rural radiography (39.3 vs 33.6/10^6^ people) and ultrasound (24.7 vs 14.5/10^6^ people) resources exceed metropolitan by 17 and 70%, respectively. Additionally, rural access to mammography is almost triple that in the metropole (14 vs 5 units/10^6^ women > 40 years) (Table 1).

The provincial, metropolitan, and rural CT:ultrasound:radiography ratios are 1:7:13, 1:5:10 and 1:15:23, respectively (Table 1).

### Imaging staff

Of the 443 provincial imaging personnel, almost 80 % (*n* = 348/443; 79%) are in the metropole and more than three-quarters (*n* = 339/443; 77%) are radiographers (Table 1).

Metropolitan personnel resources by population (*n* = 112 vs 53/10^6^ people) and equipment unit (1.7 vs 0.7/10^6^ people) are more than double the rural. Of note, the rural areas have more equipment units than personnel (Table 1).

The provincial, metropolitan and rural radiologist:sonographer:radiographer ratios are 1:1:11, 1:0.8:10 and 1:2:16 respectively (Table 1).

The potential average annual workload (excluding ultrasound) by radiologist at provincial, metropolitan and rural levels was 35,820, 30,219 and 63,825 studies, respectively (Table 1).

### Imaging service utilization

More than 1.2 million studies were performed across the modalities, averaging 262 examinations/10^3^ people and 1.3 investigations/patient. The CXR (*n* = 92/10^3^ people) was the commonest single examination, while plain radiography accounted for almost three-quarters (*n* = 935,607, 73%) of all investigations. Radiography (*n* = 935,607, 73%) and ultrasound (*n* = 202,639, 16%) together constituted almost 90 % (*n* = 1,138,246, 89%) of all provincial investigations (Table 2).

**Table 2:** WCP radiological utilization patterns

	Rural	Metropolitan	Western Cape Province (WCP)
**Studies (per 10**^**3**^**people)**	**380,657 (213.6)**	**896,587 (289.4)**	**1,277,244 (261.7)**
**Chest x-ray (n)**	132,237	315,539	447,776
per 10^3^ people	74.2	101.8	91.7
per 10^3^ patients	468.6	441.9	449.4
**General x-ray (n)**	173,292	314,539	487,831
per 10^3^ people	97.2	101.5	100.0
per 10^3^ patients	614.0	440.5	489.6
**Total x-ray (n)**	**305,529**	**630,078**	**935,607**
per 10^3^ people	171.4	203.4	191.7
per 10^3^ patients	1082.6	882.3	939.1
per equipment unit	4364.7	6058.4	5377.1
**Ultrasound (n)**	**61,534**	**141,105**	**202,639**
per 10^3^ people	34.5	45.5	41.5
per 10^3^ patients	218.0	197.6	203.4
per equipment unit	1398.5	3135.7	2276.8
**Fluoroscopy (n)**	**2247**	**14,410**	**16,657**
per 10^3^ people	1.3	4.7	3.4
per 10^3^ patients	8.0	20.2	16.7
per equipment unit	140.4	450.3	347.0
**Mammography (n)**	**1374**	**12,396**	**13,770**
per 10^3^ women > 40 years	4.7	24.3	17.2
per equipment unit	458.0	6198.0	2754.0
**CT**	**9973**	**65,877**	**75,850**
per 10^3^ people	5.6	21.3	15.5
per 10^3^ patients	35.3	92.3	76.1
per equipment unit	3324.3	6587.7	5834.6
**MR**		**12,504**	**12,504**
per 10^3^ people		4.0	2.6
per 10^3^ patients		17.5	12.6
Per equipment unit		4168.0	4168.0
**Lodox**		**4463**	**4463**
per 10^3^ people		1.4	0.9
per 10^3^ patients		6.2	4.5
Per equipment unit		1487.7	1487.7
**Angiography**		**15,754**	**15,754**
per 10^3^ people		5.1	3.2
per 10^3^ patients		22.1	15.8
Per equipment unit		1750.4	1750.4
Normal hours (% of total Studies)	299,285 (78.6)	637,447 (71.1)	936,732 (73.3)
After hours (% of total Studies)	81,372 (21.4)	259,140 (28.9)	340,512 (26.7)
**Total Studies**	**380,657 (100)**	**896,587 (100)**	**1,277,244 (100)**
x-ray and ultrasound (% of Studies)	367,063 (96.4)	771,183 (86.0)	1,138,246 (89.1)
per 10^3^ people	206.0	248.9	233.2
per 10^3^ patients	1300.6	1079.9	1142.5
Workload (Excluding Ultrasound)	319,123	755,482	1,074,605
Workload per Radiologist	63,825	30,219	35,820
Total Studies per equipment	2739	4269	3660
Ratio to Rural	1.0	1.6	1.3

Overall population-based utilization of imaging services across the modalities was 30% higher in the metropole (289 vs 214 studies/10^3^ people), with mammography (24 vs 5 studies/10^3^ woman > 40 years; differential = 517%) and CT (21 vs 6/10^3^ people; differential = 380%) recording the highest differences and plain radiography utilization (203 vs 171/10^3^ people; differential = 19%) the lowest. However, overall patient-based utilization of services in the rural areas exceeded that in the metropole by 20% (1301 vs 1080 studies/10^3^ patients) reflecting increased plain radiography and ultrasound usage (Table 2).

The provincial, metropolitan and rural CT:ultrasound:radiography ratios were 1:3:12, 1:2:10, 1:6:31, respectively (Table 2).

### Imaging equipment utilization

Provincial equipment units performed an average of 3660 studies annually. Average metropolitan equipment outputs by unit exceeded those in the rural area by more than 50 % (4269 vs 2739/unit, differential = 56%). The highest differences were in mammography (*n* = 6198 vs 458/unit) and fluoroscopy (*n* = 450 vs 140/unit). Seventy percent of all equipment usage was confined to normal working hours. This figure was similar for the province, the metropolitan and the rural areas (Table 2).

## Discussion

To our knowledge, this is the most detailed analysis to date of the usage of radiological services in either a low- or a middle-income country. It broadly reviews a provincial imaging platform, while allowing a better understanding of key differences between metropolitan and rural service provision and utilization. It therefore represents a seminal work in the field, that contributes significantly to discourses on equitable access to basic healthcare, universal health coverage and appropriate utilization of diagnostic imaging. It can serve as a benchmark resource and stimulate further work in this domain. There are two key findings in this study.

Firstly, the number of rural imaging facilities and equipment units per million people exceeds that in the metropole. This represents a commitment on the part of the WCP DoH to enhance access to imaging for the relatively sparse rural population. However, metropolitan personnel resources by population (*n* = 112 vs 53 imaging staff/10^6^ people) and equipment unit (1.7 vs 0.7 staff/unit) are more than double the rural. Furthermore, the number of rural equipment units exceeds personnel.

Secondly, notwithstanding the higher rural imaging facility and equipment resources by population, overall rural service utilization (214 studies/10^3^ people) was 30% less than metropolitan (289 studies/10^3^ people). Despite the rural areas being 17, 70 and 263% better resourced for plain radiography, ultrasound and mammography, respectively, utilization of these modalities was just 74, 45 and 7% of that in the metropole. Imaging utilization thus appears to represent a complex interplay between various determinants within the imaging enterprise. This is further illustrated by the finding that plain radiographic and ultrasound examinations *per patient* were 23 and 10% higher, respectively, in the rural areas compared to the metropole. The intuitive explanation is that this reflects lower CT availability in the rural districts (1.7 scanners/10^6^ people) compared to the metropole (3.2 scanners/10^6^ people). The inference is that if there is not ready access to CT, a more expensive modality with higher diagnostic sensitivity and specificity, recourse is made to the more basic, affordable, and accessible option. Conversely, there may also be a concurrent lower threshold for metropolitan CT utilization, given the more ready availability of the modality.

Our findings show that while steps have been taken to achieve imaging equity in the WCP, this remains a work in progress and that further interventions and iterative analyses will be required if this is to be realised.

This work also provides novel insights into the differential equipment workload across the modalities and regions. CT (*n* = 5835) and plain radiography (*n* = 5377) achieved the highest average annual outputs per unit across the province, while fluoroscopy recorded the lowest (*n* = 347), being approximately 17-fold below that of CT. The limited use of fluoroscopy reflects declining global trends and suggests that provincial policy on fluoroscopic service provision merits review. Of note, less than 3 fluoroscopic investigations/10^3^ people were performed across the province in the review period.

Rural equipment utilization by unit was less than metropolitan across all modalities. The smallest differential was in plain radiography, where the average rural unit (*n* = 4365) achieved 72% of metropolitan output (*n* = 6058), whilst the greatest difference was in mammography, with rural output (*n* = 458) a mere 7% of metropolitan (*n* = 6198). The optimal combination of equipment, personnel, and hours of operation for rural facilities remains a conundrum. WCP rural equipment resources currently exceed imaging personnel by 46% (*n* = 139 vs 95), likely contributing to decreased equipment utilization. A recent study of Zambian radiological equipment and personnel resources [25] found more than three Zambian diagnostic radiographers per equipment unit, nationally, and at least two radiographers per unit at provincial level, even in the most sparsely populated regions. Strategies to enhance rural equipment utilization could include optimizing personnel-to-equipment ratios, extending facility operating hours, as well as patient education initiatives, the latter particularly applicable to mammography services.

Even allowing for uncertainty in ultrasound outputs by personnel category, this work provides important insights into the reporting workload generated by other modalities. Excluding ultrasounds, 755,482 metropolitan and 319,123 rural studies required formal radiologist reporting, including 630,078 metropolitan and 305,529 rural plain radiographs. This translates to a potential annual workload of 63,825 and 30219studies per radiologist in the rural and metropolitan areas, respectively.

Most metropolitan radiologists have university affiliations, and thus both clinical and academic commitments, while rural consultants have exclusively clinical commitments. Of note, public sector radiologists do not have private practice commitments, so there are no competing or confounding interests. It is acknowledged that supervision of trainees impacts the clinical output of academic radiologists [32, 33]. There is thus wide acceptance that the clinical load of the academic radiologist should be capped [14, 33, 34]. The Royal Australian and New Zealand College of Radiologists (RANZCR) recommends a threshold of 12,000 examinations per year [14]. It has previously been shown that the increasing clinical workload of the WCP academic radiologist over the past decade has necessitated the prioritization of reporting of special investigations, such as fluoroscopy, CT, mammography, MRI and DSA, with resultant decreased capacity for plain radiograph reporting [35]. Unreported plain radiographs are typically interpreted informally, by non-radiologists. The average special investigation workload of the metropolitan radiologist in the review period was 5016 cases, representing a manageable annual workload when combined with selective plain radiograph reporting and educational commitments.

This is the first detailed appraisal of the potentially overwhelming workload of the rural radiologist. If all imaging studies performed in the rural areas were to be formally reported by a radiologist (*n* = 76,131) this would be more than 5-fold the average caseload (*n* = 14,900) documented for general radiology consultants working in the United States [36] and more than nine times that of general radiologists (*n* = 8171) in the United Kingdom (UK) [37]. It is clearly not a realistic expectation. In the rural areas of the WCP, the same pragmatic reporting policy applies, with mandatory special-investigation reporting, but selective plain radiograph reporting, making for a more realistic and sustainable consultant radiologist workload. The average annual special investigation workload of the individual rural radiologist in the review period was 2719 cases.

The challenge of meeting the ever-increasing demand for radiology reporting is not unique to lower resourced environments [38, 39]. A recent report showed that 97% of UK radiology departments were unable to meet all reporting requirements [38], that most delays involved plain radiograph reporting and that only 12% of National Health Service (NHS) Trusts reported all radiological examinations. There were instances where Trusts were not reporting the bulk of in-patient and emergency department plain radiographs.

To the best of our knowledge, the only comparable analysis of geographic variation in the utilization of radiographic services was conducted in Norway in 2002 [28]. The study showed that Oslo (1532.7 people/km^2^) and Finnmark (1.6 people/km^2^) were the counties with the highest and lowest population densities, respectively [40]. Plain radiographic utilization in Oslo (921 studies/10^3^ people) was double that in the more sparsely populated Finnmark (459 studies/10^3^ people). By comparison, there was a 1:1.8 differential in plain radiographic utilization between metropolitan (203 studies/10^3^ people) and rural (171 studies/10^3^ people) areas of the WCP.

The increasing population dose from medical exposures to ionising radiation is a global concern [27]. This study provides important utilization data for the WCP. This facilitates international comparisons, notwithstanding the WCP having a younger population and a higher prevalence of tuberculosis and HIV than the well-resourced countries for which comparative data are available. Utilization data from 35 European countries [27, 41] show that Hungary (*n* = 581) and Romania (*n* = 143) have the highest and lowest plain radiographic examinations per 10^3^ people, respectively, while the WCP (*n* = 192) utilization is at the lower end of this range. WCP plain radiograph utilization is most closely aligned with Sweden (*n* = 201/10^3^ people) and Denmark (*n* = 180/10^3^ people).

Our analysis also allowed comparison of WCP CT utilization with global data [27, 41]. The United States performs the highest (*n* = 271) and Costa Rica the lowest (*n* = 34) CT examinations per 10^3^ people. CT utilization in the WCP (16/10^3^ people) is less than half that of Costa Rica, also an upper middle-income country [42].

Our study provides a compelling argument for upscaling undergraduate medical education programs in the interpretation of plain radiographs and the performance of basic ultrasound examinations. Equipped with such skills, medical graduates would be well-placed to address approximately 90 % of provincial reporting needs. Additionally, competence in basic interpretation of plain radiographs would address the current reporting void in this modality. Our study also shows that educational initiatives in CXR interpretation would be particularly beneficial, since almost half of all plain radiographic studies are CXR.

This study’s major strengths are it being the first analysis of radiological utilization patterns in a middle-income country, its inclusion of all components of the so-called “imaging enterprise”, and the differentiation of rural from metropolitan services. In addition, the study focused exclusively on the utilization of public-sector resources, which provide services to the uninsured population, the more vulnerable members in our society. Published studies to date have been confined to high-income countries, have not correlated utilization with resources, and have not always drawn a clear distinction between private and public-sector resources and utilization patterns.

The study was limited by its retrospective design. However, this was mitigated by the meticulous collection and collation of all healthcare facility data in standardised format. An additional limitation was the inability to accurately assess the ultrasound workload of the various categories of personnel within the radiology domain, since outputs were not stratified by healthcare worker. In the WCP, ultrasounds in the radiological domain may be performed by either specialist radiologists, radiology registrars, sonographers, or dual-qualified radiographers, the latter performing both radiography and ultrasound. While radiologists, dual-qualified radiographers and sonographers practice independently, registrars generally report under specialist supervision. A further limitation is that many provincial obstetric and gynaecological ultrasounds, as well as point of care ultrasounds, are not performed within the radiological domain and are not included in this analysis. Going forward, WCP ultrasound data would be enhanced by allocating outputs by category of healthcare worker and including data from outside the radiological domain.

## Conclusion

The study highlights WCP attempts to bridge the divide between rural and metropolitan access to imaging, through the provision of an increased number of imaging facilities and basic equipment units per million people in the rural compared to metropolitan areas. However, it also underscores the complexity of achieving equitable utilization of services between rural and metropolitan areas.

The achievement of equity in all aspects of healthcare must be seen as a process, involving incremental improvements and iterative analyses that define progress towards the goal. Studies such as this serve to define the baseline and inform future interventions to enhance equity going forward.

## Data Availability

All radiological details were extracted from the databases of the Medical Imaging Services Sub Directorate of the Directorate of Health Technology of the WCP DoH for the public healthcare sector of the WCP of SA for 2017. The datasets used and/or analysed during the current study are available as an additional supporting file or from the corresponding author on reasonable request.

## Abbreviations

LMIC: Low- and middle-income countries
SDG: Sustainable Development Goals
WHO: World Health Organization
WCP: Western Cape Province
SA: South Africa
DHS: District Health System
DoH: Department of Health
CXR: Chest radiographs
GR: General radiographs
CT: Computed tomography
MRI: Magnetic resonance imaging
WCG: Western Cape Government
